# Supplementary material for: High Bio-Content Thermoplastic Polyurethanes from Azelaic Acid
Source: Molecules. 2022 Jul 30;27(15):4885. doi: 10.3390/molecules27154885 (PMC9370010; doi:10.3390/molecules27154885)
Supplement: Supplementary file 1 [file molecules-27-04885-s001.zip › molecules-1789750-supplementary.pdf]

High Bio-Content Thermoplastic Polyurethanes from Azelaic Acid

Bhausaheb S. Rajput, Thien An Phung Hai and Michael D. Burkart*

Department of Chemistry and Biochemistry, University of California, San Diego, 9500 CA 92093-0116 Gilman Drive, La Jolla, CA 92093-0358, USA; bsrajput@ucsd.edu (BSR); h1phung@ucsd.edu (TAPH); mburkart@ucsd.edu (MDB).

*Correspondence: mburkart@ucsd.edu.

<u>**NMR Spectra**</u>

1. Polyester-polyol AzAPDO:

^{1}H NMR (500 MHz, $CDCl_3$, δ): 4.17-4.05 (s), 3.60 (s), 2.20 (s), 1.87 (s), 1.77 (s), 1.53 (s), 1.22.
^{13}C NMR (126 MHz, $CDCl_3$, δ): 174.10-173.68 (s, C=O), 61.24-59.04 (s), 34.22 (s), 31.77 (s), 28.92 (s), 28.0 (s), 24.70 (s).

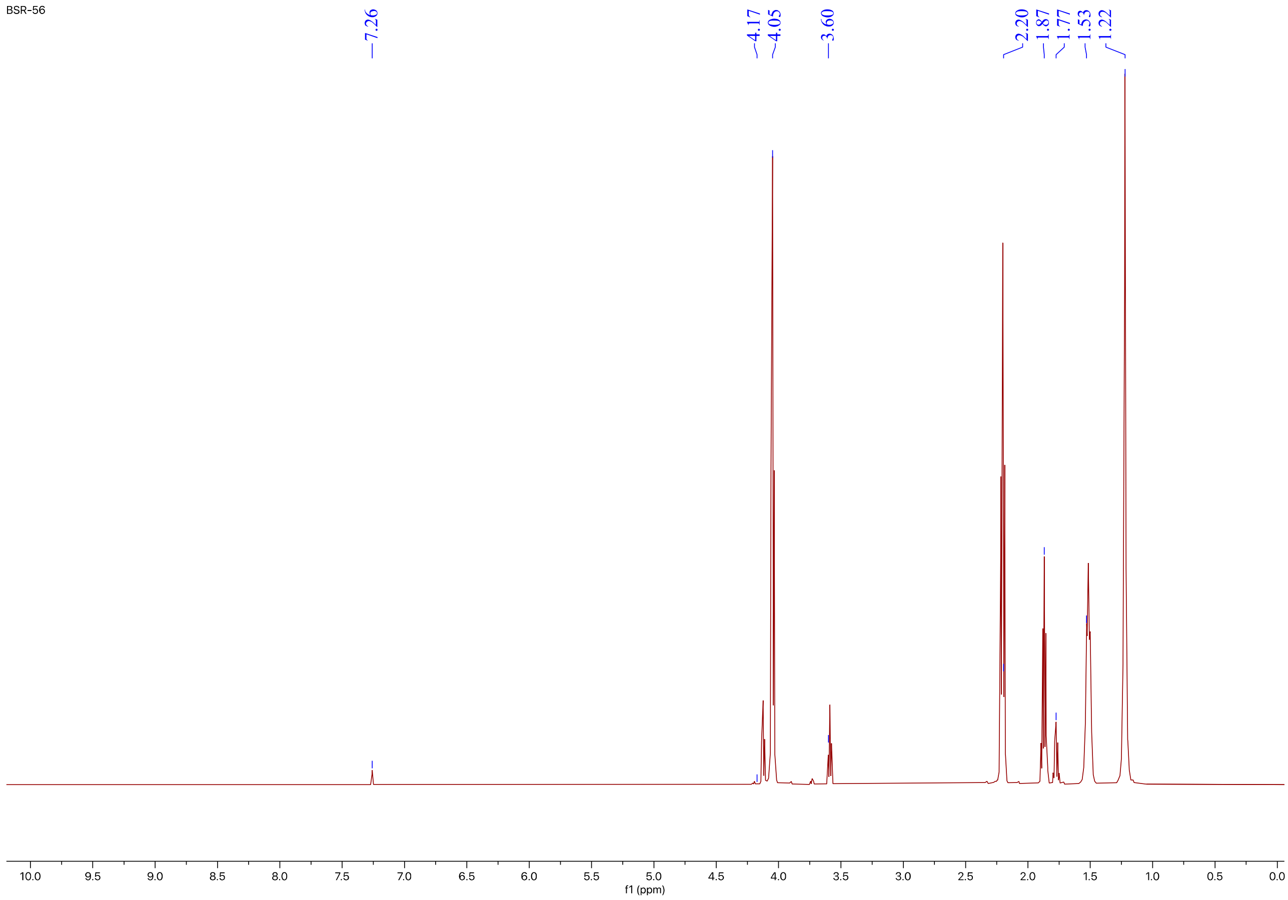

Figure S1: ^{1}H NMR of AzAPDO polyester-polyol in $CDCl_3$ (500 MHz, 298K).

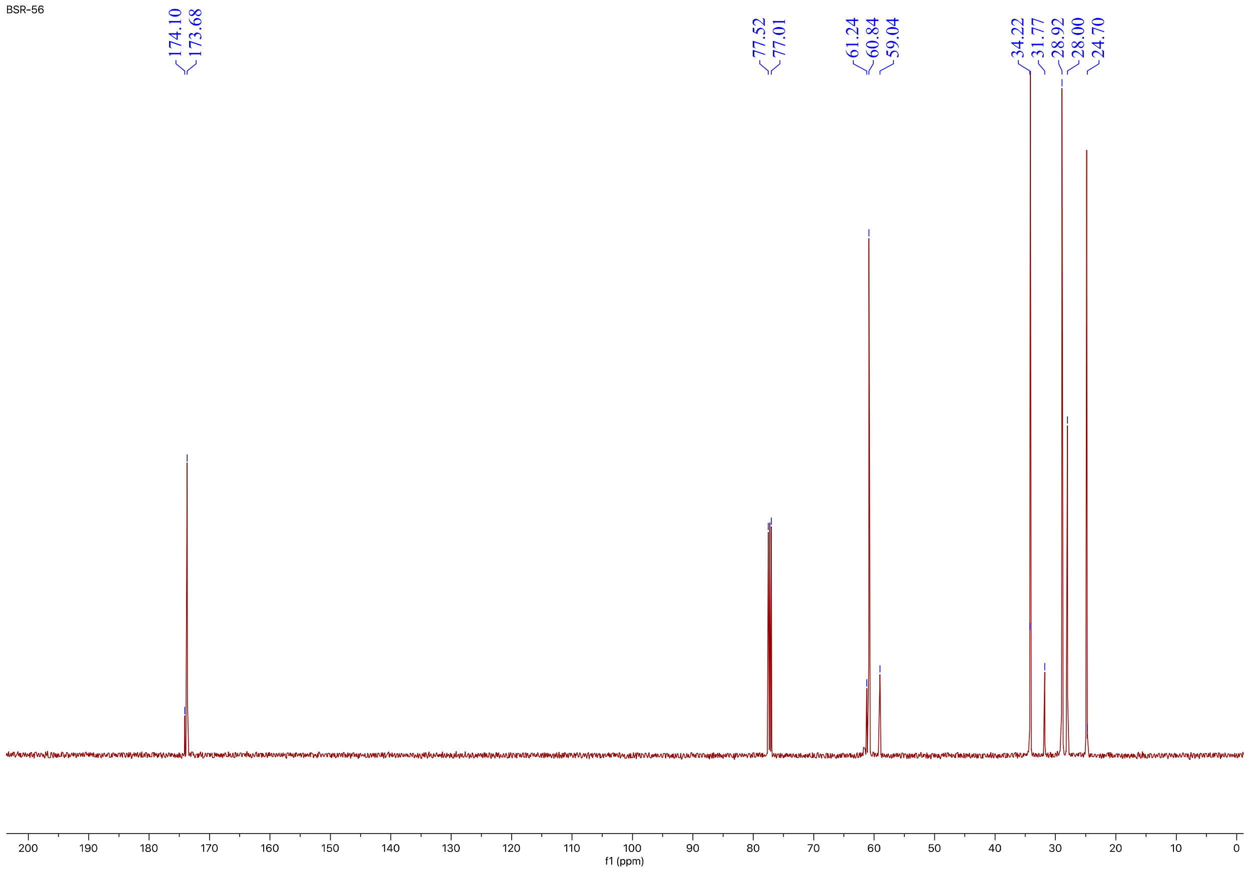

Figure S2: ^{13}C NMR of AzAPDO polyester-polyol in $CDCl_3$ (125 MHz, 298K).

2. Polyester-polyol AzA2MPDO:

^{1}H NMR (500 MHz, $CDCl_3$, δ): 4.05-3.95 (s), 3.51-3.43 (s), 2.24-2.08 (s), 1.57-1.52 (s), 1.25-1.24 (s), 0.93- 0.89(s). **^{13}C NMR** (126 MHz, $CDCl_3$, δ): 174.26-173.74 (s, C=O), 66.0-64.43 (s), 35.54 (s), 34.21 (s), 32.46 (s), 28.98 (s) 24.75 (s), 13.92 (s).

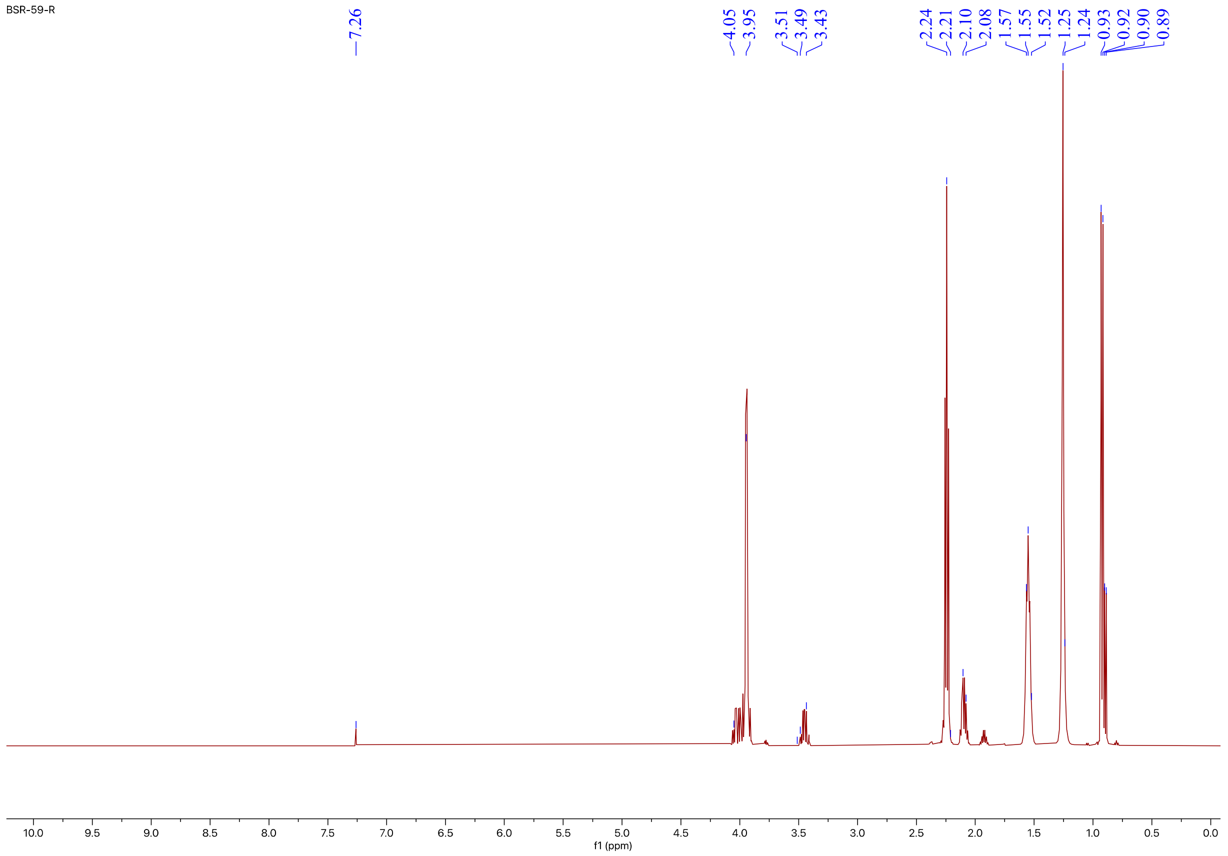

Figure S3: ^{1}H NMR of AzA2MPDO polyester-polyol in $CDCl_3$ (500 MHz, 298K).

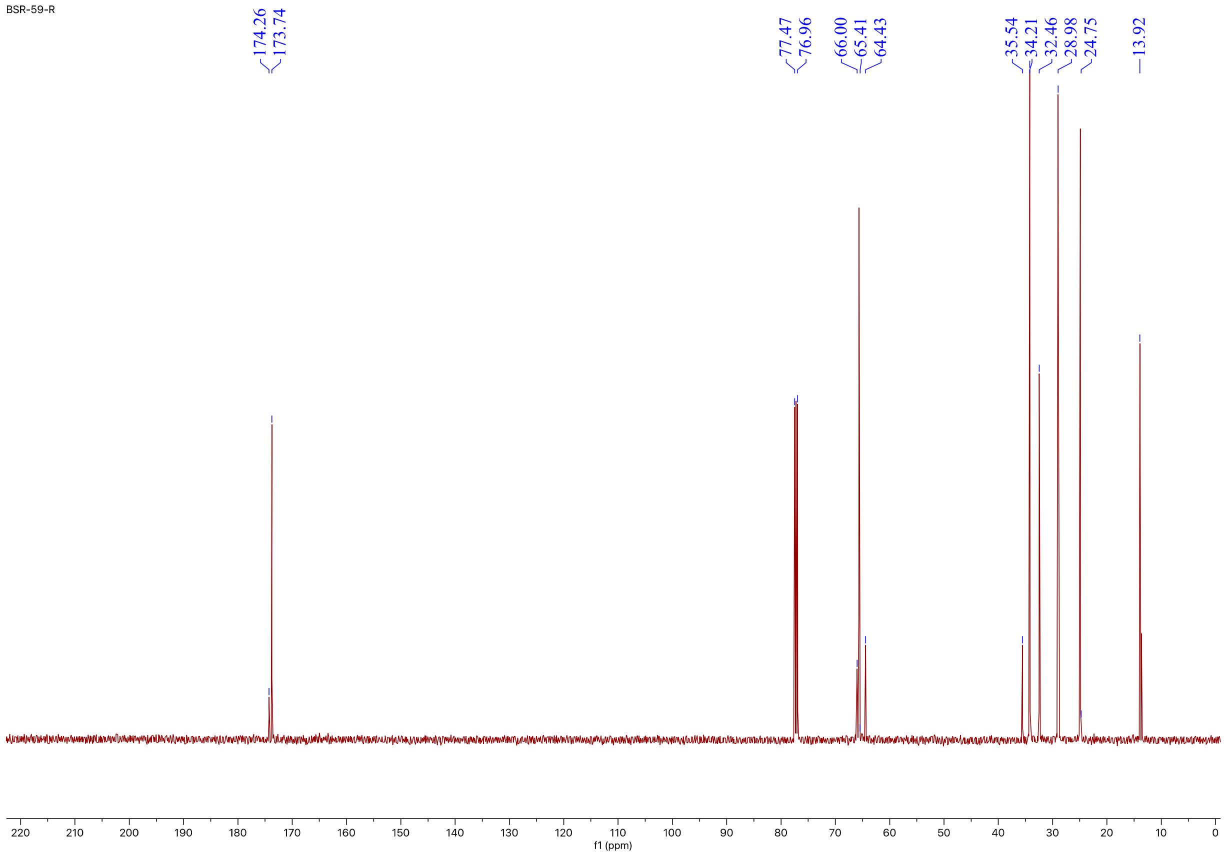

Figure S4: ^{13}C NMR of AzA2MPDO polyester-polyol in $CDCl_3$ (125 MHz, 298K).

3. Polyester-polyol AzA3MPDO:

^{1}H NMR (500 MHz, $CDCl_3$, δ): 4.08-4.02 (s), 3.59 (s), 2.24-2.23 (s), 1.66-1.43 (s), 1.25 (s), 0.90-0.87 (s). **^{13}C NMR** (126 MHz, $CDCl_3$, δ): 173.87 (s, C=O), 62.59 (s), 60.72 (s), 39.71 (s), 35.46 (s), 34.16 (s), 29.02 (s), 27.18 (s), 24.93 (s), 19.35 (s).

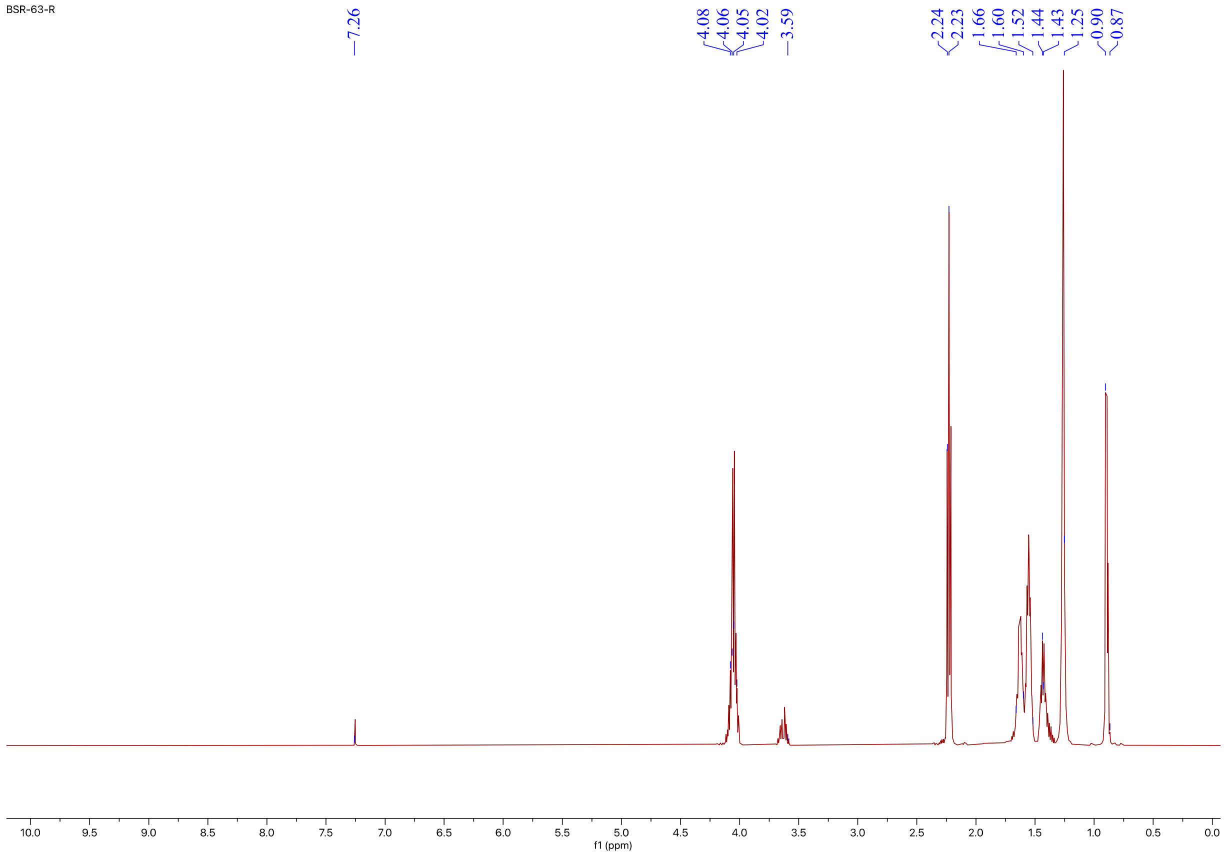

Figure S5: ^{1}H NMR of AzA3MPDO polyester-polyol in $CDCl_3$ (500 MHz, 298K).

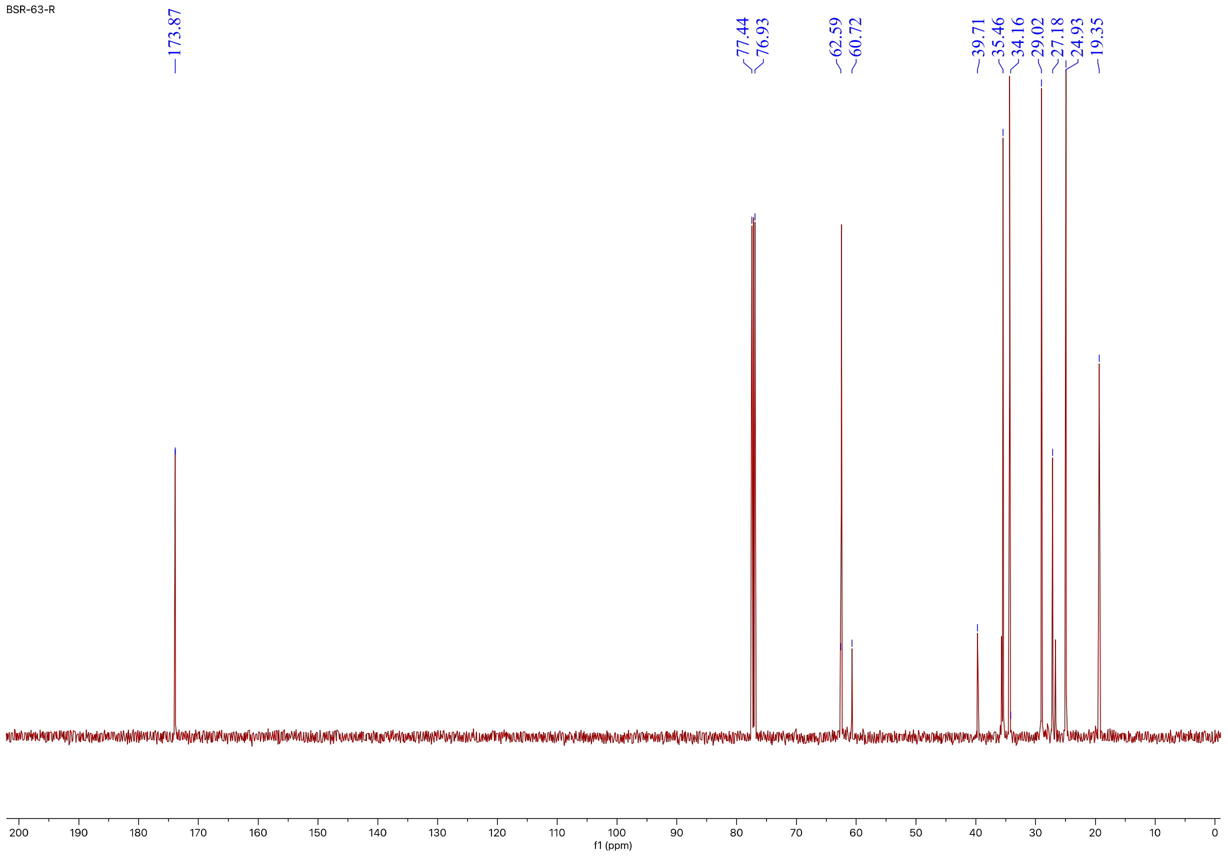

Figure S6: ^{13}C NMR of AzA3MPDO polyester-polyol in $CDCl_3$ (125 MHz, 298K).

4. Thermoplastic polyurethane TPU1:

^{1}H NMR (500 MHz, DMSO-*d6*, δ): 7.06 (urethane proton, s), 4.03-3.96 (s), 2.91 (s), 2.27 (s), 2.25 (s), 1.87 (s), 1.49 (s), 1.35 (s), 1.21 (s). **^{13}C NMR** (126 MHz, DMSO-*d6*, δ): 173.33 (s, C=O), 156.68 (urethane carbon, s), 61.06 (s), 40.49 (s), 33.90 (s), 29.89 (s), 28.85 (s), 28.08 (s), 26.47 (s), 24.88 (s).

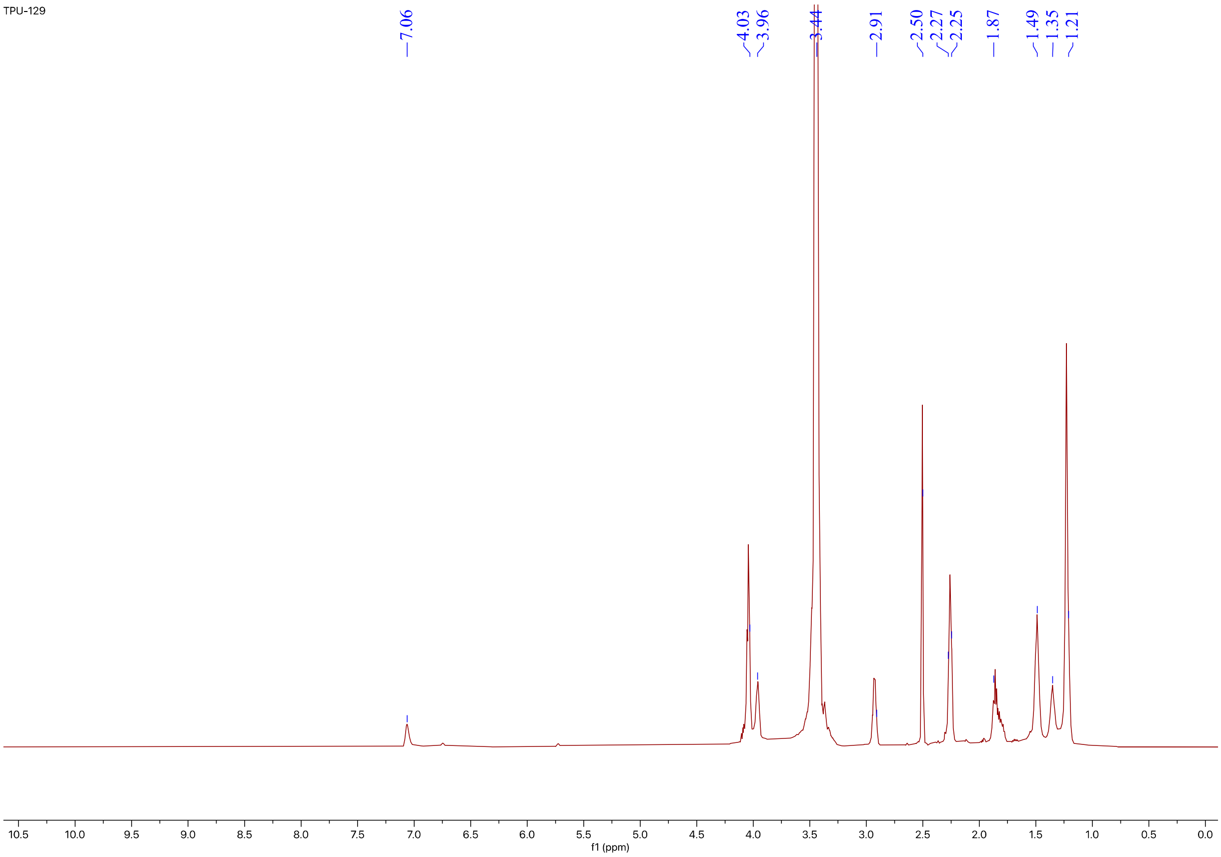

Figure S7: ^{1}H NMR of TPU1 in DMSO-d_6 (500 MHz, 298K).

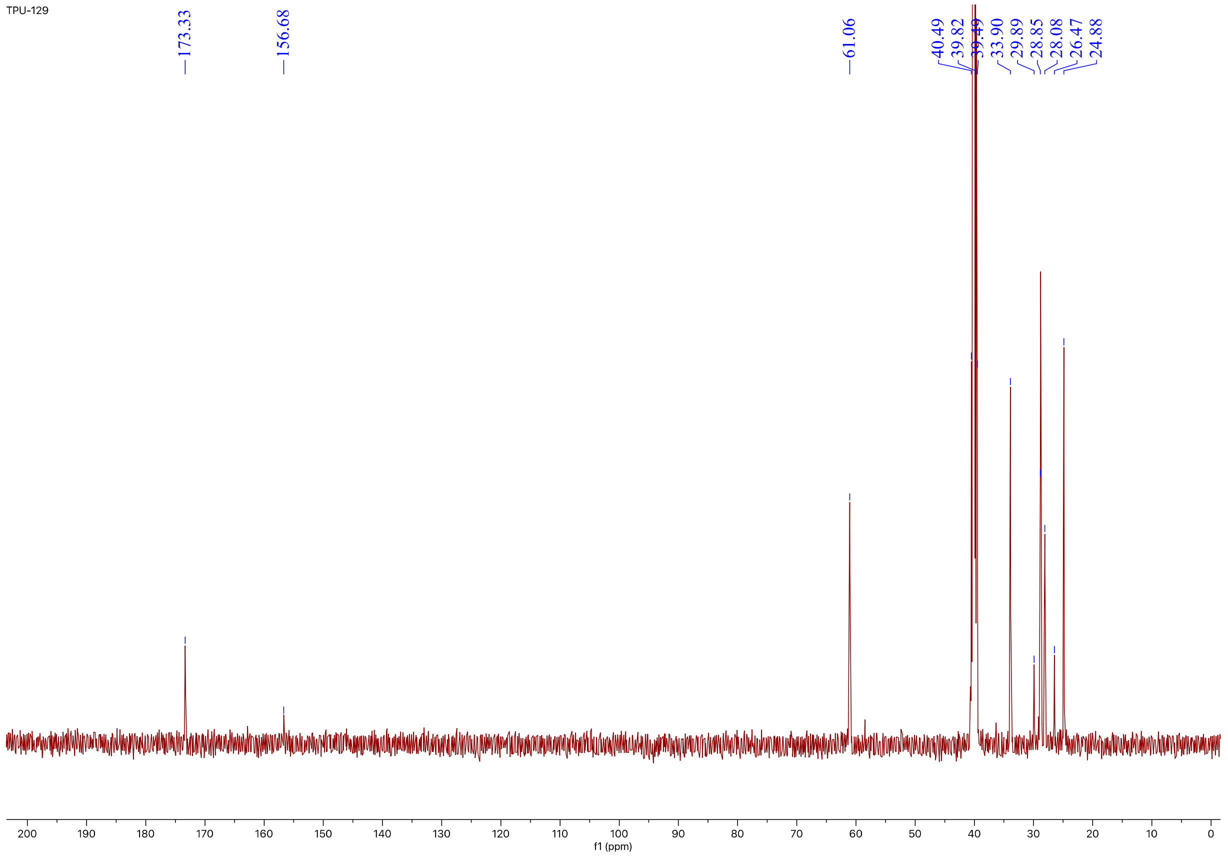

Figure S8: ^{13}C NMR of TPU1 in DMSO-*d*₆ (125 MHz, 298K).

5. Thermoplastic polyurethane TPU2:

^{1}H NMR (500 MHz, DMSO-*d6*, δ): 7.07 (urethane proton, s), 3.98-3.91 (s), 2.93-2.91 (s), 2.25 (s), 1.50 (s), 1.36 (s), 1.21 (s), 0.88-0.87 (s). **^{13}C NMR** (126 MHz, DMSO-*d6*, δ): 173.45 (s, C=O), 156.71 (urethane carbon, s), 65.72 (s), 61.0 (s), 33.73 (s), 32.61 (s), 28.68 (s), 26.62 (s), 24.79 (s), 14.05 (s).

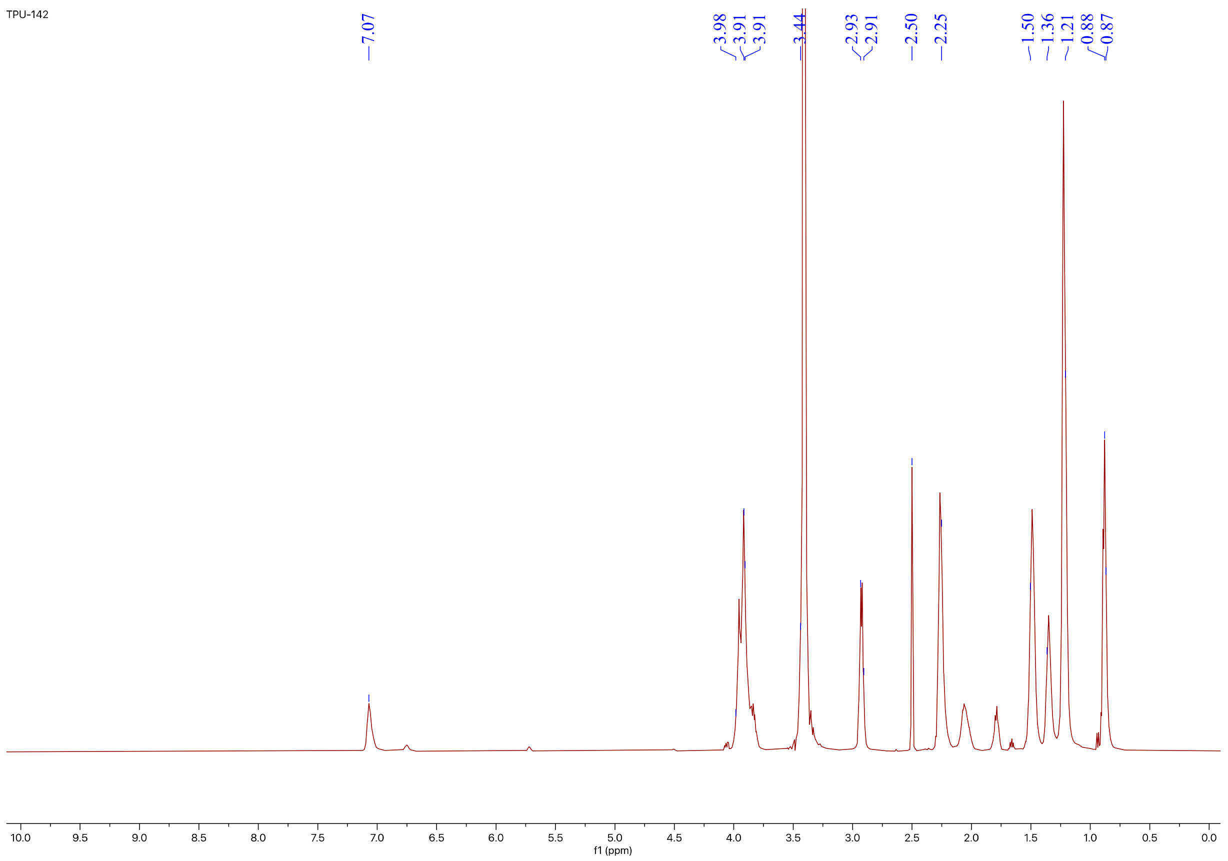

Figure S9: ^{1}H NMR of TPU2 in DMSO-d_6 (500 MHz, 298K).

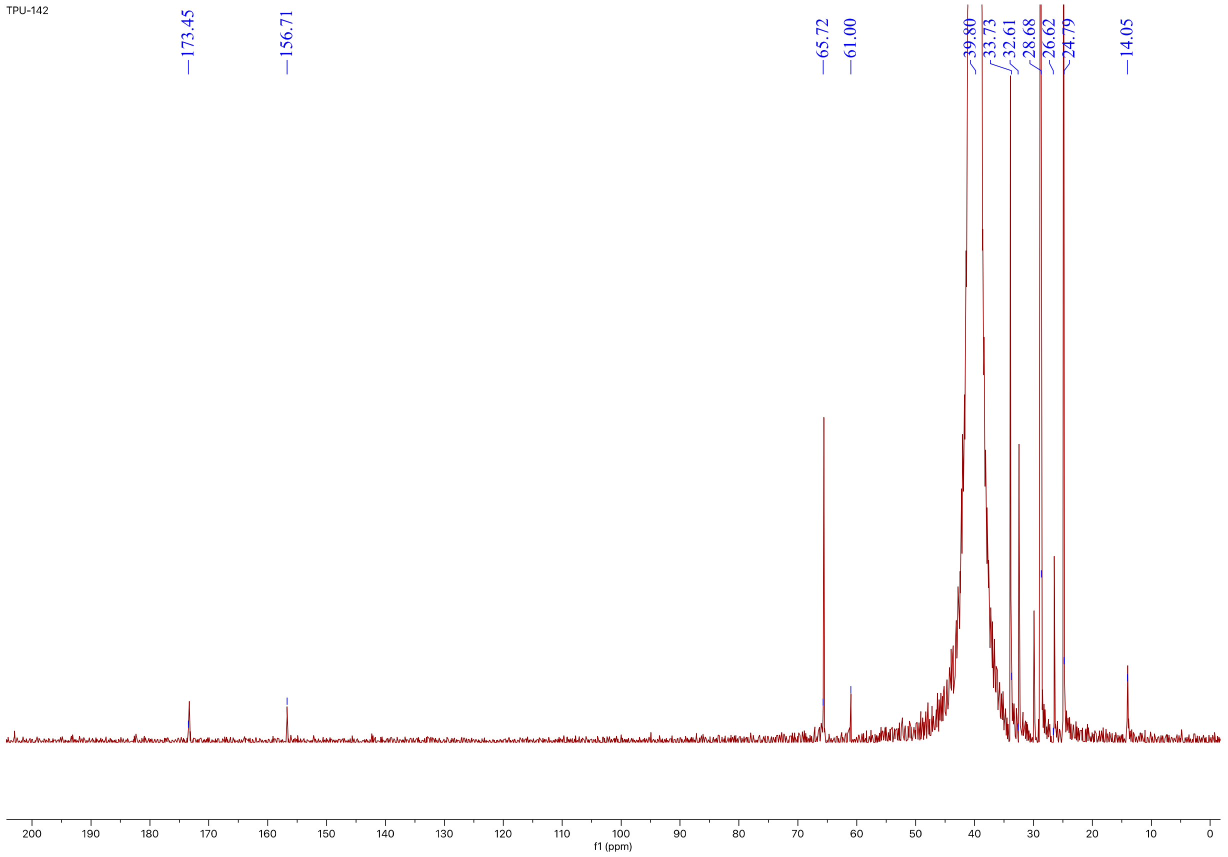

Figure S10: ^{13}C NMR of TPU2 in DMSO-d_6 (125 MHz, 298K).

6. Thermoplastic polyurethane TPU3:

^{1}H NMR (500 MHz, DMSO-*d6*, δ): 7.06-6.99 (urethane proton, s), 4.02 (s), 2.92 (s), 2.25 (s), 1.58 (s), 1.48 (s), 1.35 (s), 1.22 (s), 0.83 (s).

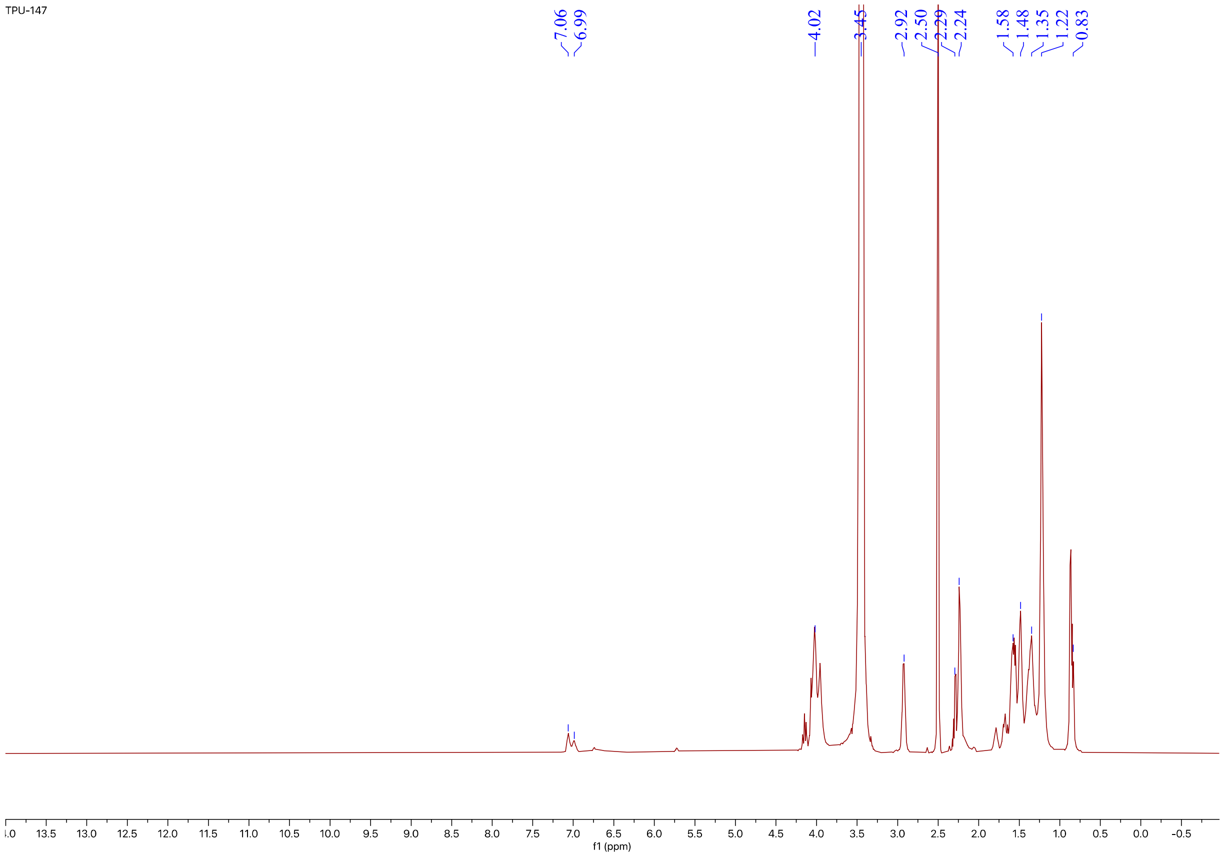

Figure S11: ^{1}H NMR of TPU3 in DMSO-d_6 (500 MHz, 298K).
